# New Formulae for the High-Order Derivatives of Some Jacobi Polynomials: An Application to Some High-Order Boundary Value Problems

**DOI:** 10.1155/2014/456501

**Published:** 2014-10-14

**Authors:** W. M. Abd-Elhameed

**Affiliations:** ^1^Department of Mathematics, Faculty of Science, King Abdulaziz University, Jeddah, Saudi Arabia; ^2^Department of Mathematics, Faculty of Science, Cairo University, Giza 12613, Egypt

## Abstract

This paper is concerned with deriving some new formulae expressing explicitly the high-order derivatives of Jacobi polynomials whose parameters difference is one or two of any degree and of any order in terms of their corresponding Jacobi polynomials. The derivatives formulae for Chebyshev polynomials of third and fourth kinds of any degree and of any order in terms of their corresponding Chebyshev polynomials are deduced as special cases. Some new reduction formulae for summing some terminating hypergeometric functions of unit argument are also deduced. As an application, and with the aid of the new introduced derivatives formulae, an algorithm for solving special sixth-order boundary value problems are implemented with the aid of applying Galerkin method. A numerical example is presented hoping to ascertain the validity and the applicability of the proposed algorithms.

## 1. Introduction

Classical orthogonal polynomials are successfully employed for solving ordinary and partial differential equations in spectral and pseudospectral methods (see, for instance, [1–3]). In particular, the class of Jacobi polynomials *P*_*n*_^(*α*,*β*)^(*x*) plays prominent roles in the applications of mathematical analysis. For example, Doha et al. in [4] used these polynomials for solving some odd-order boundary value problems (BVPs). The suggested algorithms in this paper are based on selecting certain combinations of Jacobi polynomials satisfying the boundary conditions of the given differential equation. There are some other articles in literature that have extensive theoretical and numerical studies about Jacobi polynomials (see, e.g., [5, 6]). It is well known that the classical Jacobi polynomials have two parameters. Of course, special choices of their parameters give special kinds of these polynomials. It is worthy to mention here that the classical Jacobi polynomials have six special well-known kinds of orthogonal polynomials; they are Legendre, ultraspherical, and Chebyshev polynomials of the four kinds. All six special kinds of Jacobi polynomials have their roles and importance from both theoretical and practical points of view (see, e.g., [7–11]).

The spectral methods have prominent roles in various applications such as fluid dynamics. These methods are global methods. There are three popular methods of spectral methods; they are tau, collocation, and Galerkin methods (see, e.g., [12, 13]). The choice of the suitable used spectral method suggested for solving the given equation depends certainly on the type of the differential equation and also on the type of the boundary conditions governed by it.

The explicit formulae for the high-order derivatives of various orthogonal polynomials in terms of their original polynomials are useful when spectral and pseudospectral methods are used for obtaining numerical solutions of various types of differential equations. Formula for the high-order derivatives for Chebyshev polynomials in terms of their original polynomials is given in Karageorghis [14]. The corresponding formulae for Legendre, ultraspherical, and Jacobi polynomials are given, respectively, in Phillips [15] and Doha [16].

Due to their great importance in various applications, high even-order BVPs have been investigated by a large number of authors. For example, sixth-order BVPs are known to arise in astrophysics; the narrow convecting layers bounded by stable layers, which are believed to surround A-type stars, may be modeled by sixth-order BVPs. Sixth-order BVPs were handled by numerous numerical techniques. For example, Doha and Abd-Elhameed in [17] have developed efficient solutions of multidimensional sixth-order BVPs based on employing symmetric generalized Jacobi-Galerkin method. Also, Bhrawy et al. in [18] have developed an extension of the Legendre-Galerkin method for handling sixth-order BVPs with variable polynomial coefficients. There are other contributions concerning sixth-order BVPs; among the techniques used for handling this kind of BVPs are sinc-Galerkin method [19], nonpolynomial spline technique in [20], spline collocation method in [21], Adomian decomposition method with Green's function in [22], parametric quintic spline solution in [23], homotopy perturbation method in [24], and fourth order finite difference method in [25]. For extensive studies about the existence and uniqueness of solutions of such problems, the interested reader can be refered to the important book of Agarwal [26]. For other studies in high even- and high odd-order BVPs, see, for example, [27–31].

The main objective of this paper is twofold:

- developing new formulae for the high-order derivatives of some Jacobi polynomials with certain parameters,
- employing the new introduced formulae in solving some sixth-order BVPs based on applying a spectral Galerkin method.

The rest of the paper is as follows. In the next section, some useful properties of Jacobi polynomials are presented. In Section 3, we derive two new formulae which give explicitly the high-order derivatives of Jacobi polynomials whose parameters difference is one in terms of their original Jacobi polynomials. In Section 4, we give other two new formulae which give explicitly the high-order derivatives of Jacobi polynomials whose parameters difference is two in terms of their original Jacobi polynomials. Some new reduction formulae for summing some terminating hypergeometric functions of the type _3_ *F*_2_(1) are deduced in Section 5.  Section 6 is devoted to implementing and presenting a Galerkin algorithm for numerically solving ceratin sixth-order BVPs including a numerical example aiming to illustrate the accuracy and the efficiency of the proposed algorithm. Finally, conclusions are given in Section 7.

## 2. Some Properties of Jacobi Polynomials

The classical Jacobi polynomials associated with the real parameters (*α* > −1, *β* > −1) and the weight function *w*(*x*) = (1 − *x*)^*α*^(1 + *x*)^*β*^ (see, e.g., [32, 33]) are a sequence of polynomials *P*_*j*_^(*α*,*β*)^(*x*)  (*j* = 0,1, 2,…), each, respectively, of degree *j*. These polynomials have the following Gauss hypergeometric form:

(1) Pj(α,β)(x)=(α+1)jj!F12(−j,j+λ;α+1|1−x2)=(−1)j(β+1)jj!F12(−j,j+λ;β+1|1+x2),

where

(2) $$\begin{array}{c} \lambda =\alpha +\beta +1,\;\;{\left(z\right)}_{k}=\frac{\Gamma \left(z+k\right)}{\Gamma \left(z\right)}. \end{array}$$

It is clear that

(3) $$\begin{array}{c} P_{j}^{\left(\beta ,\alpha \right)}\left(-x\right)={\left(-1\right)}^{j}P_{j}^{\left(\alpha ,\beta \right)}\left(x\right), \end{array}$$

(4) $$\begin{array}{c} P_{j}^{\left(\alpha ,\beta \right)}\left(1\right)=\frac{{\left(\alpha +1\right)}_{j}}{j!},\;\;P_{j}^{\left(\alpha ,\beta \right)}\left(-1\right)=\frac{{\left(-1\right)}^{j}{\left(\beta +1\right)}_{j}}{j!}. \end{array}$$

The Jacobi polynomials may be generated using the recurrence relation

(5) 2(j+1)(j+λ)(2j+λ−1)Pj+1(α,β)(x) =(2j+λ−1)3xPj(α,β)(x)+(α2−β2)(2j+λ)Pj(α,β)(x)  −2(j+α)(j+β)(2j+λ+1)Pj−1(α,β)(x),

starting from *P*_0_^(*α*,*β*)^(*x*) = 1 and *P*_1_^(*α*,*β*)^(*x*) = (1/2)[*α* − *β* + (*λ* + 1)*x*], or obtained alternatively from Rodrigue's formula

(6) Pj(α,β)(x)=(−1)j2jj!(1−x)−α(1+x)−β ×djdxj[(1−x)α+j(1+x)β+j].

The polynomials *P*_*j*_^(*α*,*β*)^(*x*) satisfy the orthogonality relation

(7) ∫−11(1−x)α(1+x)βPi(α,β)(x)Pj(α,β)(x)dx ={0,i≠j,hi(α,β),i=j,

where

(8) $$\begin{array}{c} h_{i}^{\left(\alpha ,\beta \right)}=\frac{2^{\alpha +\beta +1}\Gamma \left(i+\alpha +1\right)\Gamma \left(i+\beta +1\right)}{\left(2i+\alpha +\beta +1\right)i!\Gamma \left(i+\alpha +\beta +1\right)}. \end{array}$$

The following structure formula is useful in the sequel (see Rainville [34]):

(9) Pj(α,β)(x)=2(j+λ−1)(2j+λ−1)3 ×[(j+λ−1)2(2j+λ−1)DPj+1(α,β)(x)  +(α−β)(j+λ−1)(2j+λ)DPj(α,β)(x)  −(j+α)(j+β)(2j+λ+1)DPj−1(α,β)(x)],                     j≥1.

Also, the following theorem is of important use hereafter.

**Theorem 1 .**

For all *q* ≥ 1, the *q*th derivative of the Jacobi polynomial *P*_*j*_^(*α*,*β*)^(*x*) is given explicitly by

(10) $$\begin{array}{c} D^{q}P_{j}^{\left(\alpha ,\beta \right)}\left(x\right)=\overset{j-q}{\underset{i=0}{\sum }}\theta_{i,j,\alpha ,\beta ,q}P_{i}^{\left(\alpha ,\beta \right)}\left(x\right), \end{array}$$

where

(11) θi,j,α,β,q=(j+λ)q(j+q+λ)i(i+q+α+1)j−i−qΓ(i+λ)2q(j−i−q)!Γ(2i+λ) ×F23(−j+q+i,j+i+q+λ,    i+α+1;i+q+α+1,2i+λ+1 ∣ 1).

(For a proof of Theorem 1, see Doha [16].)

**Remark 2 .**

Although the _3_ *F*_2_(1) in formula (11) is terminating, it cannot be summed in a closed form except for certain special choices of the two parameters *α* and *β*. In case of *α* = *β*, this _3_ *F*_2_(1) can be summed in a closed form with the aid of Watson's identity (see Doha [16]).

## 3. High-Order Derivatives of *P*_*n*_^(*α*,*α*+1)^(*x*) and *P*_*n*_^(*α*+1,*α*)^(*x*)

The main objective of this section is to state and prove two theorems in which the derivatives of the Jacobi polynomials *P*_*n*_^(*α*,*α*+1)^(*x*) and *P*_*n*_^(*α*+1,*α*)^(*x*) are given in terms of their corresponding Jacobi polynomials. We first state and prove a lemma in which the first derivative of *P*_*n*_^(*α*,*α*+1)^(*x*) is expressed in terms of their original polynomials.

**Lemma 3 .**

For all *n* ≥ 1, one has

(12) DPn(α,α+1)(x)=∑r=0⌊(n−1)/2⌋dn,rPn−2r−1(α,α+1)(x) +∑r=0⌊n/2⌋−1en,rPn−2r−2(α,α+1)(x),

where

(13) dn,r=(2n−2r+2α+1)Γ(n+α+1)Γ(n−2r+2α+1)Γ(n+2α+2)Γ(n−2r+α),en,r=2(r+1)Γ(n+α+1)Γ(n−2r+2α)Γ(n+2α+2)Γ(n−2r+α−1),

and ⌊*z*⌋ denotes the largest integer less than or equal to *z*.

**Proof**

Let us denote

(14) $$\begin{array}{c} S_{m}\left(x\right)=\overset{m}{\underset{n=0}{\sum }}a_{n}P_{n}^{\left(\alpha ,\alpha +1\right)}\left(x\right), \end{array}$$

(15) $$\begin{array}{c} I_{m+1}\left(x\right)=\int S_{m}\left(x\right)dx. \end{array}$$

The integration of both sides of the structure formula (9) (for the case *β* = *α* + 1) yields

(16) ∫Pn(α,α+1)(x)dx=αnPn+1(α,α+1)(x)+βnPn(α,α+1)(x) +γnPn−1(α,α+1)(x), n≥1,

where

(17) αn=n+2α+2(n+α+1)(2n+2α+3),βn=−2(2n+2α+1)(2n+2α+3),γn=−(n+α)(n+2α+1)(2n+2α+1),

which in turn implies that

(18) Im+1(x)=α0∫P0(α,α+1)(x)dx+∑n=1man[αnPn+1(α,α+1)(x)+βnPn(α,α+1)(x)+γnPn−1(α,α+1)(x)].

The last equation may be written alternatively in the form

(19) $$\begin{array}{c} I_{m+1}\left(x\right)=\overset{m+1}{\underset{n=0}{\sum }}A_{n}P_{n}^{\left(\alpha ,\alpha +1\right)}\left(x\right), \end{array}$$

where

(20) an−1αn−1+anβn+γn+1an+1=An,n=m+1,m,…,1,am+1=am+2=0.

The difference equation (20) can be solved to give

(21) an=∑k=n+1(k+n)  oddm+1gk,nAk+∑k=n+2(k+n)  evenm+1fk,nAk,n=0,1,…,m,

where

(22) gk,n=(k+n+2α+2)Γ(k+α+1)Γ(n+2α+2)Γ(k+2α+2)Γ(n+α+1),

(23) fk,n=(k−n)Γ(k+α+1)Γ(n+2α+2)Γ(k+2α+2)Γ(n+α+1).

If we substitute (21) into (14), we get

(24) Sm(x) =∑n=0m{∑k=n+1(k+n)  oddm+1gk,nAk+∑k=n+2(k+n)  evenm+1fk,nAk,}Pn(α,α+1)(x).

On the other hand, if we differentiate (19) with respect to *x*, we have

(25) $$\begin{array}{c} S_{m}\left(x\right)=\overset{m+1}{\underset{n=0}{\sum }}A_{n}DP_{n}^{\left(\alpha ,\alpha +1\right)}\left(x\right). \end{array}$$

Equating the two right-hand sides of the two relations (24) and (25) yields

(26) ∑n=0m{∑k=n+1(k+n)  oddm+1gk,nAk+∑k=n+2(k+n)  evenm+1fk,nAk,}Pn(α,α+1)(x) =∑n=0m+1AnDPn(α,a+1)(x).

Expanding the left-hand side of (26) and collecting the similar terms, (26) may be written—after some rather manipulation—in the form

(27) $$\begin{array}{c} \overset{m+1}{\underset{n=0}{\sum }}A_{n}B_{n}\left(x\right)=\overset{m+1}{\underset{n=0}{\sum }}A_{n}DP_{n}^{\left(\alpha ,a+1\right)}\left(x\right), \end{array}$$

where

(28) Bn(x)=∑r=0⌊(n−1)/2⌋gn,n−2r−1Pn−2r−1(α,α+1)(x) +∑r=0⌊n/2⌋−1fn,n−2r−2Pn−2r−2(α,α+1)(x),

and *g*_*k*,*n*_, *f*_*k*,*n*_ are given by (22) and (23), respectively. This immediately yields

(29) DPn(α,α+1)(x)=∑r=0⌊(n−1)/2⌋dn,rPn−2r−1(α,α+1)(x) +∑r=0⌊n/2⌋−1en,rPn−2r−2(α,α+1)(x),

where

(30) dn,r=(2n−2r+2α+1)Γ(n+α+1)Γ(n−2r+2α+1)Γ(n+2α+2)Γ(n−2r+α),en,r=2(r+1)Γ(n+α+1)Γ(n−2r+2α)Γ(n+2α+2)Γ(n−2r+α−1),

and this completes the proof of of Lemma 3.

Now, we extend the result of Lemma 3 to give the *q*th-derivative of the Jacobi polynomials *P*_*n*_^(*α*,*α*+1)^(*x*) in terms of their original polynomials. The general formula is stated in the following theorem.

**Theorem 4 .**

For all *q* ≥ 1, the *q*th-derivative of the classical Jacobi polynomials *P*_*n*_^(*α*,*α*+1)^(*x*) of any degree and for any order in terms of their corresponding Jacobi polynomials is given explicitly by

(31) DqPn(α,α+1)(x)=∑r=0⌊(n−q)/2⌋An,r,qPn−2r−q(α,α+1)(x) +∑r=0⌊(n−q−1)/2⌋Bn,r,qPn−2r−q−1(α,α+1)(x),

where

(32) An,r,q=(2q(q+r−1)!Γ(n+α+1)Γ(n−r+α+32) ×Γ(n−q−2r+2α+2)) ×((q−1)!r!Γ(n+2α+2)Γ(n−q−2r+α+1)   ×Γ(n−q−r+α+32))−1,

(33) Bn,r,q=(2q(q+r)!Γ(n+α+1)Γ(n−r+α+12) ×Γ(n−q−2r+2α+1)) ×((q−1)!r!Γ(n+2α+2)Γ(n−q−2r+α)   × Γ(n−q−r+α+32))−1.

**Proof**

We proceed by induction on *q*. For *q* = 1, Theorem 4 reduces identically to Lemma 3. Assume that relation (31) holds, and we have to show that

(34) Dq+1Pn(α,α+1)(x)=∑r=0⌊(n−q−1)/2⌋An,r,q+1Pn−2r−q−1(α,α+1)(x) +∑r=0⌊(n−q)/2⌋−1Bn,r,q+1Pn−2r−q−2(α,α+1)(x).

If we differentiate relation (31), then we get

(35) Dq+1Pn(α,α+1)(x)=∑r=0⌊(n−q)/2⌋An,r,qDPn−2r−q(α,α+1)(x) +∑r=0⌊(n−q−1)/2⌋Bn,r,qDPn−2r−q−1(α,α+1)(x),

and in virtue of Lemma 3, one can write

(36) $$\begin{array}{c} D^{q+1}P_{n}^{\left(\alpha ,\alpha +1\right)}\left(x\right)=\underset{1}{\sum }+\underset{2}{\sum }, \end{array}$$

where

(37) ∑1=∑r=0⌊(n−q)/2⌋An,r,q∑s=0⌊(n−q−1)/2⌋−rdn−q−2r,sPn−q−2r−2s−1(α,α+1)(x) +∑r=0⌊(n−q−1)/2⌋Bn,r,q∑s=0⌊(n−q−1)/2⌋−r−1en−q−2r−1,sPn−q−2r−2s−3(α,α+1)(x),∑2=∑r=0⌊(n−q)/2⌋An,r,q∑s=0⌊(n−q)/2⌋−r−1en−q−2r,sPn−q−2r−2s−2(α,α+1)(x) +∑r=0⌊(n−q−1)/2⌋Bn,r,q∑s=0⌊(n−q)/2⌋−r−1dn−q−2r−1,sPn−q−2r−2s−2(α,α+1)(x).

If we expand ∑_1_ and ∑_2_ and collecting similar terms and after some lengthy manipulations, we get

(38) Dq+1Pn(α,α+1)(x)=∑r=0⌊(n−q−1)/2⌋A¯n,r,qPn−2r−q−1(α,α+1)(x) +∑r=0⌊(n−q)/2⌋−1B¯n,r,qPn−2r−q−2(α,α+1)(x),

where

(39) A¯n,r,q=∑j=0r{An,j,qdn−q−2j,r−j+Bn,j,qen−q−2j−1,r−j−1},B¯n,r,q=∑j=0r{An,j,qen−q−2j,r−j+Bn,j,qdn−q−2j−1,r−j}.

It is not difficult to show that

(40) A¯n,r,q=(2q−1(2n−2r+2α+1)Γ(n+α+1) × Γ(n−q−2r+2α+1)) ×((q−1)!Γ(n+2α+2)Γ(n−q−2r+α))−1 ×∑j=0r(((j+q−1)!(2n−4j−2q+2α+1)     × Γ(n−j+α+12))    × (j!Γ(n−j−q+α+32))−1),B¯n,r,q=(2q(q+r+1)Γ(n+α+1) × Γ(n−q−2r+2α)) ×((q−1)!Γ(n+2α+2)Γ(n−q−2r+α−1))−1 ×∑j=0r(((j+q−1)!(2n−4j−2q+2α+1)     × Γ(n−j+α+12))    × (j!Γ(n−j−q+α+32))−1).

To complete the proof of Theorem 4, the following lemma—which can be easily proved by induction—is required.

**Lemma** **5.** *For every nonnegative integer r* *, one has*

(41) ∑j=0r(j+q−1)!(2n−4j−2q+2α+1)Γ(n−j+α+1/2)j!Γ(n−j−q+α+3/2) =2(q+r)!Γ(n−r+α+1/2)qr!Γ(n−q−r+α+1/2).

Now, the application of Lemma 5 in (40) yields

(42) A−n,r,q=(2q+1(q+r)!Γ(n+α+1)Γ(n−r+α+32)  × Γ(n−q−2r+2α+1)) ×(q!r!Γ(n+2α+2)Γ(n−q−2r+α)   × Γ(n−q−r+α+12))−1=An,r,q+1,B−n,r,q=(2q+1(q+r+1)!Γ(n+α+1)Γ(n−r+α+12)  ×Γ(n−q−2r+2α)) ×(q!r!Γ(n+2α+2)Γ(n−q−2r+α−1)   × Γ(n−q−r+α+12))−1=Br,n,q+1.

This proves formula (34) and hence completes the proof of Theorem 4.

**Remark 6 .**

It is worthy to note here that relation (31) may be written alternatively in the equivalent form

(43) $$\begin{array}{c} D^{q}P_{n}^{\left(\alpha ,\alpha +1\right)}\left(x\right)=\overset{n-q}{\underset{i=0}{\sum }}E_{n,i,q}P_{i}^{\left(\alpha ,\alpha +1\right)}\left(x\right), \end{array}$$

where

(44) En,i,q=2qΓ(n+α+1)Γ(i+2α+2)(q−1)!Γ(i+α+1)Γ(n+2α+2) ×{((n−i+q−2)/2)!Γ((n+i+q+2α+3)/2)((n−i−q)/2)!Γ((n+i−q+2α+3)/2),            (n+i+q)  even,((n−i+q−1)/2)!Γ((n+i+q+2α+2)/2)((n−i−q−1)/2)!Γ((n+i−q+2α+4)/2),            (n+i+q)  odd.

**Remark 7 .**

As a direct consequence of relation (3), the *q*th-derivative of *P*_*n*_^(*α*+1,*α*)^(*x*) is given by

(45) DqPn(α+1,α)(x)=∑r=0⌊(n−q)/2⌋An,r,qPn−2r−q(α+1,α)(x) −∑r=0⌊(n−q−1)/2⌋Bn,r,qPn−2r−q−1(α+1,α)(x),

where *A*_*n*,*r*,*q*_ and *B*_*n*,*r*,*q*_ are given by (32) and (33), respectively, or alternatively in the form

(46) $$\begin{array}{c} D^{q}P_{n}^{\left(\alpha +1,\alpha \right)}\left(x\right)=\overset{n-q}{\underset{i=0}{\sum }}{\left(-1\right)}^{n+i+q}E_{n,i,q}P_{i}^{\left(\alpha +1,\alpha \right)}\left(x\right), \end{array}$$

where *E*_*n*,*i*,*q*_ is given by (44).

As a special case of the two formulae (31) and (45) and if we set *α* = −1/2, the derivatives of Chebyshev polynomials of third kind (*V*_*n*_(*x*)) and of fourth kind (*W*_*n*_(*x*)) can be easily deduced. These two results are given in the following two corollaries.

**Corollary 8 .**

For all *q* ≥ 1, the *q*th-derivative of *V*_*n*_(*x*) of any degree and for any order in terms of their corresponding polynomials is given explicitly by

(47) DqVn(x)=2q(q−1)!∑r=0⌊(n−q)/2⌋(n−r)!(r+q−1)!r!(n−r−q)!Vn−2r−q(x) +2q(q−1)! ×∑r=0⌊(n−q−1)/2⌋(n−r−1)!(r+q)!r!(n−r−q)!Vn−2r−q−1(x).

**Corollary 9 .**

For all *q* ≥ 1, the *q*th-order derivative of *W*_*n*_(*x*) of any degree and for any order in terms of their corresponding polynomials is given explicitly by

(48) DqWn(x)=2q(q−1)!∑r=0⌊(n−q)/2⌋(n−r)!(r+q−1)!r!(n−r−q)!Wn−2r−q(x) −2q(q−1)! ×∑r=0⌊(n−q−1)/2⌋(n−r−1)!(r+q)!r!(n−r−q)!Wn−2r−q−1(x).

## 4. High-Order Derivatives of *P*_*n*_^(*α*,*α*+2)^(*x*) and *P*_*n*_^(*α*+2,*α*)^(*x*)

In this section, and following similar procedures to those followed in Section 3, we can obtain new derivatives formulae for the high-order derivatives of the Jacobi polynomials *P*_*n*_^(*α*,*α*+2)^(*x*) and *P*_*n*_^(*α*+2,*α*)^(*x*) in terms of their corresponding Jacobi polynomials. The details of the required computations are lengthy and will not be given here.

**Theorem 10 .**

For all *q* ≥ 1, the *q*th-derivative of the classical Jacobi polynomials *P*_*n*_^(*α*,*α*+2)^(*x*) of any degree and for any order in terms of their corresponding Jacobi polynomials is given explicitly by

(49) DqPn(α,α+2)(x)=∑r=0⌊(n−q)/2⌋Gn,r,qPn−2r−q(α,α+2)(x) +∑r=0⌊(n−q−1)/2⌋Hn,r,qPn−2r−q−1(α,α+2)(x),

where

(50) Gn,r,q =(2−q−2r(q+r−1)!Γ(n+α+1)Γ(2n−2r+2α+3))  ×((q−1)!r!Γ(n+2α+3)(n−q−2r+α+2)   ×Γ(n−r+α+2))−1  ×Γ(n−q−2r+2α+3)Γ(n−q−2r+α+5/2)Γ(2n−2q−4r+2α+3)Γ(n−q−r+α+5/2)  ×(n2−n(q+2r−2α−3)−α(q+2r)   +2r(q+r)−q−3r+α2+3α+2),

(51) Hn,r,q =(2−q−2r(q+r)!Γ(n+α+1)Γ(2(n−r+α+1)))  ×((q−1)!r!Γ(n+2α+3)(n−q−2r+α+1)   × Γ(n−r+α+1))−1  ×Γ(n−q−2r+α+3/2)Γ(n−q−2r+2α+2)Γ(n−q−r+α+3/2)Γ(2n−2q−4r+2α+1).

**Remark 11 .**

As a direct consequence of relation (49), the *q*th-derivative of *P*_*n*_^(*α*+2,*α*)^(*x*) is given by

(52) DqPn(α+2,α)(x)=∑r=0⌊(n−q)/2⌋Gn,r,qPn−2r−q(α+2,α)(x) −∑r=0⌊(n−q−1)/2⌋Hn,r,qPn−2r−q−1(α+2,α)(x),

where *G*_*n*,*r*,*q*_ and *H*_*n*,*r*,*q*_ are given by (50) and (51), respectively.

## 5. Reduction Formulae for Some Terminating Hypergeometric Functions of the Type _3_ *F*_2_(1)

In this section, four new reduction formulae for the _3_ *F*_2_(1) that appears in relation (11) are deduced for certain choices of the two parameters *α* and *β*. These formulae are given in the following four corollaries.

**Corollary 12 .**

For all *i*, *n*, *q* ∈ *Z* ^≥0^ and *i* ≤ *n* − *q*, one has

(53) F23(−n+i+q,n+i+q+2α+2,  i+α+1;i+q+α+1,2i+2α+3 ∣ 1)=Γ(i+α+3/2)Γ(i+q+α+1)π(q−1)! ×{((n−i+q−2)/2)!Γ((n−i−q+1)/2)Γ((n+i+q+2α+2)/2)Γ((n+i−q+2α+3)/2),              (n+i+q) even,((n−i+q−1)/2)!Γ((n−i−q+2)/2)Γ((n+i+q+2α+3)/2)Γ((n+i−q+2α+4)/2),               (n+i+q) odd,F23(−n+i+q,n+i+q+2α,  i+α+1;i+q+α+1,2i+2α+1 ∣ 1)=Γ(i+α+1/2)Γ(i+q+α+1)π(q−1)!(n+α) ×{((n−i+q−2)/2)!Γ((n−i−q+1)/2)Γ((n+i+q+2α)/2)Γ((n+i−q+2α+1)/2),              (n+i+q) even,−((n−i+q−1)/2)!Γ((n−i−q+2)/2)Γ((n+i+q+2α+1)/2)Γ((n+i−q+2α+2)/2),               (n+i+q) odd.

**Corollary 13 .**

For all *i*, *n*, *q* ∈ *Z* ^≥0^ and *i* ≤ *n* − *q*, one has

(54) F23(−n+i+q,n+i+q+2α+3,  i+α+1;i+q+α+1,2i+2α+4 ∣ 1)=2−2(i+α+2)Γ(2i+2α+4)Γ(i+q+α+1)(q−1)!Γ(i+α+3) ×{((n−i+q−2)/2)!Γ((n−i−q+1)/2)Γ((n+i−q+2α+5)/2)Γ((n+i+q+2α+4)/2) × ξi,n,α,              (n+i+q) even,4((n−i+q−1)/2)!Γ((n−i−q+2)/2)Γ((n+i−q+2α+4)/2)Γ((n+i+q+2α+3)/2),              (n+i+q) odd,F23(−n+i+q,n+i+q+2α−1,  i+α+1;i+q+α+1,2i+2α ∣ 1)=Γ(i+α+1/2)Γ(i+q+α+1)2π(q−1)!(i+α)(n+α−1)(n+α) ×{((n−i+q−2)/2)!Γ((n−i−q+1)/2)Γ((n+i−q+2α+1)/2)Γ((n+i+q+2α)/2) × ηi,n,α,            (n+i+q) even,−4((n−i+q−1)/2)!Γ((n−i−q+2)/2)Γ((n+i−q+2α)/2)Γ((n+i+q+2α−1)/2),             (n+i+q) odd,

where

(55) ξi,n,α=i2+2α(i+n+3)+3i+n2+3n−q2+q+2α2+4,ηn,i,α=i2+2α(i+n−1)−i+n2−n−q2+q+2α2.

**Proof**

The proofs of the four reduction formulae in Corollaries 12 and 13 can be deduced immediately, by comparing the four relations (43), (46), (49), and (52) with the corresponding results obtained from the general relation in (10).

## 6. Jacobi Galerkin Algorithms for Sixth-Order Two-Point Boundary Value Problems

In this section, and as an application, we are interested in applying the introduced derivatives formulae which were obtained in Sections 3 and 4, for the sake of solving the following sixth-order two-point BVPs:

(56) $$\begin{array}{c} -y^{\left(6\right)}\left(x\right)+\gamma y\left(x\right)=f\left(x\right),\;x\in \left(-1,1\right), \end{array}$$

governed by the nonhomogeneous boundary conditions

(57) $$\begin{array}{c} y^{\left(r\right)}\left(\pm 1\right)=\pm \alpha_{r},\;r=0,1,2, \end{array}$$

where *γ* is a real constant.

We draw the reader's attention that (56) governed by the nonhomogeneous boundary conditions (57) can be easily transformed to the equation (see [17])

(58) $$\begin{array}{c} -u^{\left(6\right)}\left(x\right)+\gamma u\left(x\right)=g\left(x\right),\;x\in \left(-1,1\right), \end{array}$$

governed by the homogeneous boundary conditions

(59) $$\begin{array}{c} u^{\left(r\right)}\left(\pm 1\right)=0,\;r=0,1,2, \end{array}$$

where

(60) $$\begin{array}{c} g\left(x\right)=f\left(x\right)+\overset{5}{\underset{i=0}{\sum }}\eta_{i}x^{i}, \end{array}$$

and *η*_*i*_, 0 ≤ *i* ≤ 2*n* − 1, are some constants.

Now, we define the following spaces:

(61) $$\begin{array}{c} S_{N}=\mathrm{span}\left\{P_{0}^{\left(\alpha ,\beta \right)}\left(x\right),P_{1}^{\left(\alpha ,\beta \right)}\left(x\right),P_{2}^{\left(\alpha ,\beta \right)}\left(x\right),\ldots ,P_{N}^{\left(\alpha ,\beta \right)}\left(x\right)\right\},\\ V_{N}=\left\{v\in S_{N}:D^{j}v\left(\pm 1\right)=0,j=0,1,2\right\}. \end{array}$$

The Jacobi-Galerkin procedure for solving (56) subject to the boundary conditions (57) is to find *u*_*N*_ ∈ *V*_*N*_ such that

(62) $$\begin{array}{c} {\left(-D^{6}u_{N},v\right)}_{w}+\gamma {\left(u_{N},v\right)}_{w}={\left(g,v\right)}_{w},\;\forall v\in V_{N}, \end{array}$$

where *w* = (1 − *x*)^*α*^(1 + *x*)^*β*^, and (*u*, *v*)_*w*_ = ∫_−1_^1^ *wuv* *dx* is the scalar inner product in the weighted space *L*_*w*_^2^(−1,1).

### 6.1. Basis Functions in Terms of Certain Parameters Jacobi Polynomials

In this section, we consider four kinds of basis functions satisfying the boundary conditions (59). Assume that these basis functions can be expressed in the following forms:

(63) $$\begin{array}{c} \phi_{1,k}=P_{k}^{\left(\alpha ,\alpha +1\right)}\left(x\right)+\overset{6}{\underset{m=1}{\sum }}d_{1,m,k}P_{k+m}^{\left(\alpha ,\alpha +1\right)}\left(x\right),\\ \phi_{2,k}=P_{k}^{\left(\alpha +1,\alpha \right)}\left(x\right)+\overset{6}{\underset{m=1}{\sum }}d_{2,m,k}P_{k+m}^{\left(\alpha +1,\alpha \right)}\left(x\right),\\ \phi_{3,k}=P_{k}^{\left(\alpha ,\alpha +2\right)}\left(x\right)+\overset{6}{\underset{m=1}{\sum }}d_{3,m,k}P_{k+m}^{\left(\alpha ,\alpha +2\right)}\left(x\right),\\ \phi_{4,k}=P_{k}^{\left(\alpha +2,\alpha \right)}\left(x\right)+\overset{6}{\underset{m=1}{\sum }}d_{4,m,k}P_{k+m}^{\left(\alpha +2,\alpha \right)}\left(x\right), \end{array}$$

and *k* = 0,1, 2,…, *N* − 6.

The coefficients {*d*_*i*,*m*,*k*_}, 1 ≤ *i* ≤ 4, are uniquely determined such that each member of *ϕ*_*i*,*k*_(*x*), 1 ≤ *i* ≤ 4, satisfies the boundary conditions (57). For example, the coefficients {*d*_1,*m*,*k*_} are given explicitly by

(64) d1,m,k={(−1)m/2Γ(α+1)(3m/2)(k+m)!(k+α+3/2)m/2Γ(k+m+α+1)(k+α+9/2)m/2,                  m=2,4,6,((−1)(m−1)/2(m−7)Γ(α+1)(3(m−1)/2) × Γ(k+m+1)(k+α+3/2)(m−1)/2) ×(2Γ(k+m+α+1)(k+α+9/2)(m+1)/2)−1,                  m=1,3,5.

Now, the four variational formulations corresponding to the four choices of the basis functions can be written as

(65) $$\begin{array}{c} {\left(-D^{6}u_{i,N},\phi_{i,k}\right)}_{w_{i}}+\gamma {\left(u_{i,N},\phi_{i,k}\right)}_{w_{i}}={\left(g,\phi_{i,k}\right)}_{w_{i}},\;1\leq i\leq 4, \end{array}$$

where

(66) $$\begin{array}{c} w_{1}={\left(1-x\right)}^{\alpha }{\left(1+x\right)}^{\alpha +1},\;\;w_{2}={\left(1-x\right)}^{\alpha }{\left(1+x\right)}^{\alpha -1},\\ w_{3}={\left(1-x\right)}^{\alpha }{\left(1+x\right)}^{\alpha +2},\;\;w_{4}={\left(1-x\right)}^{\alpha }{\left(1+x\right)}^{\alpha -2},\\ u_{i,N}=\overset{N-6}{\underset{r=0}{\sum }}c_{i,r}\phi_{i,r}. \end{array}$$

For 1 ≤ *i* ≤ 4, let us denote

(67) $$\begin{array}{c} g_{i,k}={\left(g,\phi_{i,k}\right)}_{w_{i}},\;\;g_{i}={\left(g_{i,0},g_{i,1},\ldots ,g_{i,N-6}\right)}^{T},\\ A_{i}={\left(a_{kj}^{i}\right)}_{0\leq k,j\leq N-6},\;\;B_{i}={\left(b_{kj}^{i}\right)}_{0\leq k,j\leq N-6},\\ c_{i}={\left(c_{i,0},c_{i,1},\ldots ,c_{i,N-6}\right)}^{T}, \end{array}$$

and then (65) is equivalent to the following four matrix systems:

(68) $$\begin{array}{c} \left(A_{i}+\gamma B_{i}\right)c_{i}=g_{i}, \end{array}$$

where the nonzero elements of the matrices *A*_*i*_ and *B*_*i*_, 1 ≤ *i* ≤ 4, can be given explicitly.

Now, we give a numerical example to show the applicability and the efficiency of the proposed algorithm.

**Example 14 .**

Consider the following sixth-order linear boundary value problem (see El-Gamel et al. [19], Akram and Siddiqi [20], and Lamnii et al. [21]):

(69) $$\begin{array}{c} y^{\left(6\right)}\left(t\right)-y\left(t\right)=-6e^{t},\;t\in \left[0,1\right],\\ y\left(0\right)=1,\;\;y^{\prime }\left(0\right)=0,\;\;y^{\prime \prime }\left(0\right)=-1,\\ y\left(1\right)=0,\;\;y^{\prime }\left(1\right)=-e,\;\;y^{\prime \prime }\left(1\right)=-2e. \end{array}$$

The analytic solution to (69) is (1 − *t*)*e* ^*t*^.

The transformation *t* = (1 + *x*)/2 turns (69) into

(70) $$\begin{array}{c} u^{\left(6\right)}\left(x\right)-u\left(x\right)=-6e^{\left(x+1\right)/2},\;x\in \left[-1,1\right],\\ u\left(-1\right)=1,\;\;u^{\prime }\left(-1\right)=0,\;\;u^{\prime \prime }\left(-1\right)=-1,\\ u\left(1\right)=0,\;\;u^{\prime }\left(1\right)=-e,\;\;u^{\prime \prime }\left(1\right)=-2e, \end{array}$$

with the analytic solution *u*(*x*) = (1/2)(1 − *x*)*e* ^(1 + *x*)/2^.

In Table 1, we denote *E* = |*u* − *u*_*N*_| by the absolute errors resulting from the application of the Jacobi Galekin method (JGM) for problem (70) for various values of the parameters *α*, *β*, and *N*. Moreover, Table 2 displays a comparison between the best errors resulting from the application of JGM with the results obtained by applying the following three methods:

- sinc-Galerkin method developed in [19],
- nonpolynomial spline technique developed in [20],
- spline collocation method developed in [21].

The results in Table 2 indicate that our method is more accurate if compared with all of the above-mentioned methods.

## 7. Conclusions

This paper is concerned with introducing four new analytical formulae for the *q*th-derivative of certain parameters Jacobi polynomials in terms of their corresponding Jacobi polynomials. Moreover, some new reduction formulae for summing some terminating hypergeometric functions of unit argument are deduced. As an application, and with the aid of the new introduced derivatives formulae, some spectral solutions of a special sixth-order boundary value problem are presented. To the best of our knowledge, all the presented theoretical formulae in this paper are completely new. Moreover, we do believe that these new introduced formulae can be applied for solving other kinds of high-order boundary value problems.

## Figures and Tables

**Table 1:** Maximum absolute error *E* for *N* = 8, 12, 16.

*N*	α	β	*E*
8	$-\frac{1}{2}$	$\frac{1}{2}$	1.5451*·*10^−8^
	$\frac{1}{2}$	$-\frac{1}{2}$	1.497*·*10^−8^
	0	1	2.72189*·*10^−8^
	1	0	2.71475*·*10^−8^
	$-\frac{1}{2}$	$\frac{3}{2}$	8.1876*·*10^−8^
	$\frac{3}{2}$	$-\frac{1}{2}$	7.6352*·*10^−8^
	0	2	9.35247*·*10^−8^
	2	0	8.87656*·*10^−8^

12	$-\frac{1}{2}$	$\frac{1}{2}$	1.03683*·*10^−14^
	$\frac{1}{2}$	$-\frac{1}{2}$	1.03251*·*10^−14^
	0	1	2.56597*·*10^−14^
	1	0	2.56661*·*10^−14^
	$-\frac{1}{2}$	$\frac{3}{2}$	9.67494*·*10^−14^
	$\frac{3}{2}$	$-\frac{1}{2}$	9.25758*·*10^−14^
	0	2	1.43156*·*10^−13^
	2	0	1.37795*·*10^−13^

16	$-\frac{1}{2}$	$\frac{1}{2}$	3.15286*·*10^−16^
	$\frac{1}{2}$	$-\frac{1}{2}$	3.1637*·*10^−16^
	0	1	2.57166*·*10^−16^
	1	0	2.83627*·*10^−16^
	$-\frac{1}{2}$	$\frac{3}{2}$	2.4723*·*10^−16^
	$\frac{3}{2}$	$-\frac{1}{2}$	3.38054*·*10^−16^
	0	2	5.22802*·*10^−16^
	2	0	3.47378*·*10^−16^

**Table 2:** Comparison between the best errors of different methods.

Error	JGM	Method in [19]	Method in [20]	Method in [21]
*E*	2.4723*·*10^−16^	1.00*·*10^−4^	1.70*·*10^−7^	2.370*·*10^−9^
